# The Link Between High Sensitivity Troponin T Levels and Outcomes Among Patients with Congestive Heart Failure: A Systematic Review and Meta-Analysis Study 

**DOI:** 10.30699/ijp.2025.2057561.3440

**Published:** 2025-07-01

**Authors:** Alireza Abdollahi, Zohreh Nozarian, Samaneh Salarvand, Elham Pourebrahimi

**Affiliations:** 1 Department of Pathology, School of Medicine, Tehran University of Medical Sciences, Tehran, Iran.; 2 Department of Pathology, Farabi Eye Hospital, Tehran University of Medical Sciences, Tehran, Iran

**Keywords:** High-Sensitivity Troponin T, Congestive Heart Failure, Mortality, Hospitalization, Clinical Outcomes

## Abstract

**Background & Objective:**

High-sensitivity cardiac troponin T (hs-cTnT) has emerged as a critical biomarker in cardiovascular diseases, particularly in congestive heart failure (CHF). This systematic review and meta-analysis aimed to assess the association between hs-cTnT levels and clinical outcomes in patients with CHF.

**Methods:**

A comprehensive literature search was performed across multiple databases to identify studies evaluating the relationship between hs-cTnT levels and clinical outcomes in CHF. Eligible studies reported hazard ratios (HRs) or odds ratios (ORs) for all-cause mortality, cardiovascular mortality, or cardiovascular hospitalization.

**Results:**

Elevated hs-cTnT levels were significantly associated with adverse outcomes. The pooled HR and OR for all-cause mortality were 1.70 (95% CI, 1.49–1.94) and 6.19 (95% CI, 3.88–9.86), respectively. For cardiovascular mortality, the pooled HR was 1.59 (95% CI, 1.38–1.83) and the pooled OR was 6.87 (95% CI, 3.93–12.01). For cardiovascular hospitalization, the pooled HR was 1.56 (95% CI, 1.42–1.70) and the pooled OR was 4.32 (95% CI, 2.22–8.39).

**Conclusion:**

Elevated hs-cTnT levels are strongly associated with an increased risk of all-cause mortality, cardiovascular mortality, and cardiovascular hospitalization in patients with CHF. These findings highlight the prognostic value of hs-cTnT in the clinical management of heart failure.

## Introduction

Congestive heart failure (CHF) is a major public health concern, affecting millions of individuals worldwide and contributing substantially to morbidity, mortality, and healthcare expenditures. It is a complex clinical syndrome resulting from structural or functional cardiac abnormalities that impair the heart’s ability to maintain adequate cardiac output, leading to congestion in the pulmonary and systemic circulation (1,2). CHF may arise from diverse etiologies, including ischemic heart disease, hypertension, valvular heart disorders, and cardiomyopathies—each contributing to myocardial dysfunction and diminished ventricular performance. The global burden of CHF continues to grow, driven by an aging population, increasing prevalence of cardiovascular risk factors such as diabetes mellitus and obesity, and improved survival following acute cardiac events. Despite therapeutic advances, CHF remains a progressive and life-threatening condition, highlighting the need for early identification of high-risk patients to enhance treatment strategies and improve outcomes (3–5).

Biomarkers play a pivotal role in the diagnosis, risk stratification, and prognostication of patients with CHF. Among them, cardiac troponins specifically troponin T (TnT) and troponin I (TnI) have gained widespread recognition for their ability to detect myocardial injury. The development of high-sensitivity troponin T (hs-TnT) assays has significantly advanced cardiovascular diagnostics, allowing for the detection of even minimal myocardial damage that may not be evident through imaging or clinical assessment (6–8). Numerous studies have demonstrated that elevated hs-TnT levels in CHF patients are independently associated with adverse clinical outcomes, including hospitalization and mortality. For example, one study reported a twofold increase in mortality risk among CHF patients with detectable hs-TnT levels compared to those with undetectable levels. Additionally, findings from the PARADIGM-HF trial revealed that elevated hs-TnT levels were independently associated with worsening heart failure and all-cause mortality (9–11). These observations underscore the prognostic value of hs-TnT in CHF and support its potential utility in guiding disease monitoring and therapeutic decision-making.

However, despite robust evidence linking elevated troponin levels with poor outcomes in CHF, several challenges limit the widespread adoption of hs-TnT as a standardized prognostic tool. Variability in assay sensitivity, patient characteristics, and coexisting conditions complicate the interpretation of results and the establishment of universal cutoff values (12–14). Although the prognostic significance of hs-TnT is well recognized, consensus is still lacking on how best to integrate it into routine clinical workflows for risk stratification and treatment optimization.

## Materials and methods

This systematic review and meta-analysis adhered to the Preferred Reporting Items for Systematic Reviews and Meta-Analyses (PRISMA) guidelines.

### Systematic Search

A comprehensive search strategy was conducted across major databases, including Web of Science, Scopus, and PubMed, encompassing all available records up to February 2025. The search utilized relevant Medical Subject Headings (MeSH) terms and keywords specific to hs-cTnT in patients with congestive heart failure. The search terms focused on ("prognosis" OR "death" OR "mortality" OR "hospitalization") AND ("troponin" OR "hs-cTnT") AND ("congestive heart failure" OR "chronic heart failure").

### Inclusion and Eligibility Criteria

Eligibility criteria were established based on the PICO framework. The population (P) included adult patients with congestive heart failure. The intervention (I) was the measurement of hs-TnT levels. The comparison (C) involved patients with higher versus lower hs-TnT levels. The outcomes (O) included clinical endpoints such as mortality, hospitalization rates, and cardiovascular events. Studies were excluded if they were animal studies, case reports, focused on conditions other than CHF, lacked clear hs-TnT measurement protocols, or provided insufficient outcome data. Non-clinical studies, including laboratory-based or in vitro research, were also excluded. Furthermore, studies that focused on other cardiac troponins such as troponin I or troponin C, or that did not aim to predict survival outcomes, were not included.

### Data Extraction and Outcome Measures

Data extraction was performed independently by two authors using a standardized data collection form. Any discrepancies were resolved by consulting a third author. The extracted data included study authors, year of publication, study design, sample size, patient demographics, hs-TnT levels, follow-up duration, and clinical outcomes such as all-cause mortality, cardiovascular mortality, hospitalization rates, and cardiovascular events, along with their corresponding confidence intervals.

### Statistical Analysis and Data Synthesis

Pooled estimates for all-cause mortality, cardiovascular mortality, and cardiovascular hospitalization in CHF patients with elevated hs-cTnT levels were calculated using a random-effects model. Effect sizes for each outcome were estimated using Hedges' g for quantitative comparisons. Pooled rates and 95% confidence intervals (CIs) were determined using the *meta* package in R. Heterogeneity across studies was assessed using the I² statistic to evaluate between-study variability. Effect sizes were pooled using logarithmic transformation (PLOGIT) and analyzed using random-effects models to account for differences among studies. Funnel plots were generated to evaluate potential publication bias. All statistical analyses, including the generation of forest and funnel plots, were performed using R (R Foundation for Statistical Computing, Vienna, Austria) and RStudio (RStudio Inc., Boston, MA).

## Results

**Figure 1:**
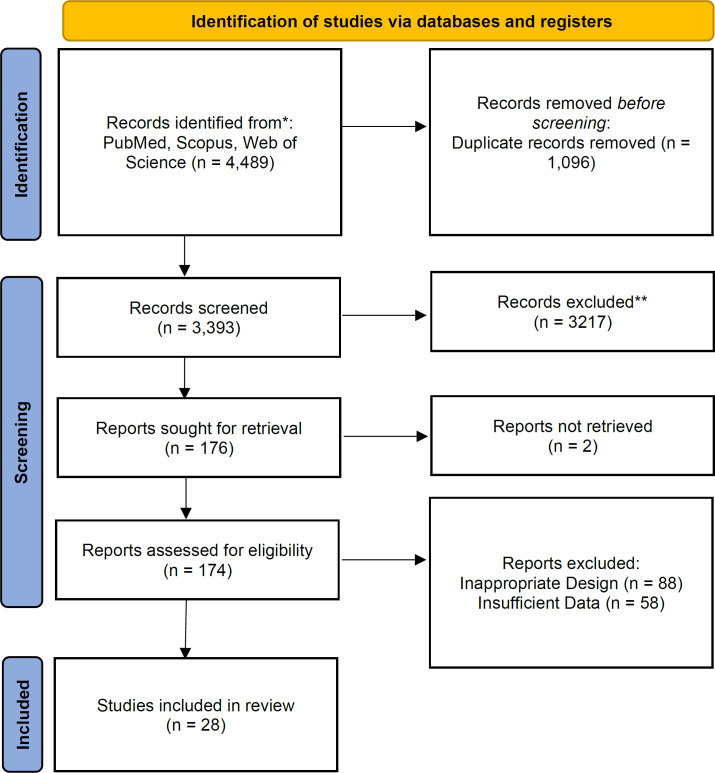
PRISMA flow diagram of the included studies

Our initial search yielded 4,489 articles from PubMed, Scopus, and Web of Science, from which we eliminated 1,096 duplicates. After reviewing the titles and abstracts of the remaining 3,393 records, we retrieved 174 full-text articles for further evaluation. Ultimately, 28 studies (6, 15-41) met our eligibility criteria and were included in the systematic review and meta-analysis (Figure 1). Detailed characteristics of the included studies are summarized in Table 1.

### All-Cause Mortality

Across the included studies, a total of 10,861 patients had low hs-cTnT levels and 10,427 had high hs-cTnT levels. Among those with low hs-cTnT, 810 deaths occurred, corresponding to a mortality rate of 7.4%, whereas 2,694 deaths were reported among those with high hs-cTnT, yielding a mortality rate of 25.8%. The pooled hazard ratio (HR) for all-cause mortality, estimated using a random-effects model, was 1.70 (95% CI, 1.49–1.94). The overall effect was statistically significant for both the common effect (z = 27.79, p < 0.01) and random-effects models (z = 8.01, p < 0.01). There was substantial heterogeneity among studies, as indicated by an I² value of 93%, τ² of 0.06, and a p value < 0.01.

Additionally, the pooled odds ratio (OR) for all-cause mortality based on the random-effects model was 3.84 (95% CI, 2.27–6.49). This effect was also significant under both the common effect model (z = 35.52, p < 0.01) and the random-effects model (z = 5.03, p < 0.01). Heterogeneity remained high, with I² = 97%, τ² = 0.58, and p < 0.01.

### Cardiovascular Mortality

For cardiovascular mortality, 10,423 patients were classified with low hs-cTnT levels and 11,513 with high hs-cTnT levels. In the low hs-cTnT group, 229 patients (2.2%) died of cardiovascular causes, compared with 1,482 patients (12.8%) in the high hs-cTnT group. The pooled hazard ratio for cardiovascular mortality using a random-effects model was 1.59 (95% CI, 1.38–1.83). The test for overall effect was statistically significant in both the common effect model (z = 6.83, p < 0.01) and the random-effects model (z = 6.40, p < 0.01). Considerable heterogeneity was observed (I² = 94%, τ² = 0.06, p < 0.01).

The pooled analysis for cardiovascular mortality using a random effects model resulted in an odds ratio of 6.87 (95% CI: 3.93, 12.01). The test for overall effect was significant (p<0.01) for both the common effect (z=23.21) and random effects models (z=6.87). The studies have high heterogeneity (I²=92%, τ²=0.34, and p<0.01).

### Cardiovascular Hospitalization

Based on the included studies, 6,201 patients had low hs-cTnT and 10,308 had high hs-cTnT. Among those with low hs-cTnT, 24.2% (1,501 cases) experienced cardiovascular hospitalization, compared to 33.3% (3,433 cases) among those with high hs-cTnT. The pooled analysis for cardiovascular hospitalization using a random effects model resulted in a hazard ratio of 1.56 (95% CI: 1.42, 1.70). The test for overall effect was significant (p<0.01) for both the common effect (z=16.92) and random effects models (z=9.68). There is moderate heterogeneity among the studies (I²=52%, τ²=0.008, and p=0.02). The pooled analysis for cardiovascular hospitalization using a random effects model resulted in an odds ratio of 4.32 (95% CI: 2.22, 8.39). The test for overall effect was significant (p < 0.01) for both the common effect (z = 22.64) and random effects models (z = 11.78). There is moderate heterogeneity among the studies (I²=69%, τ²=0.04, and p<0.01).

**Table 1:** Detailed Characteristics of the included studies

**Author**	**Year**	**Country**	**Design**	**N**	**All-cause Mortality**			**CV-Mortality**			**CV-Hospitalization**		
					**HZ**	**L**	**U**	**HZ**	**L**	**U**	**HZ**	**L**	**U**
Aimo et al. (15)	2019	Italy	PCS	1449	1.6	1.11	2.33	-	-	-	-	-	-
Aimo et al. (16)	2020	Spain	PCS	5301	1.49	1.36	1.62	1.48	1.32	1.59	1.62	1.41	1.85
Alonso et al. (17)	2016	Spain	PCS	1069	1.32	1.08	1.63	1.53	1.13	2.05	-	-	-
Berge et al. (19)	2021	Norway	PCS	314	1.56	1.34	1.8	-	-	-	-	-	-
Berg et al. (18)	2022	USA	RCT	3112	2.42	1.54	3.81	3.17	1.84	5.46	2.31	1.55	3.42
Chuang et al. (20)	2021	Australia	PCS	55000	2.03	1.92	2.15	-	-	-	1.46	1.37	1.56
Costacou et al. (21)	2020	USA	PCS	581	-	-	-	-	-	-	1.38	1.16	1.66
Eggers et al. (22)	2011	Sweden	PCS	103	-	-	-	-	-	-	-	-	-
Egstrup et al. (23)	2012	Denmark	PCS	416	1.7	1.2	2.5	-	-	-	-	-	-
Emdin et al. (24)	2018	Italy	RCS	-	1.37	1.24	1.52	1.36	1.21	1.53	1.41	1.27	1.57
Gaggin et al. (25)	2014	USA	PCS	151	-	-	-	1.11	1.03	1.19	-	-	-
Gori et al. (26)	2021	Italy	RCT	1260	-	-	-	1.34	1.14	1.57	1.43	1.11	1.85
Gravning et al. (27)	2014	Norway	RCT	1078	1.63	1.2	2.23	1.67	1.18	2.36	1.39	1.07	1.8
Januzzi et al. (28)	2022	USA	RCT	5986	-	-	-	1.03	0.77	1.37	1.68	1.09	2.27
Masson et al. (30)	2012	Italy	RCT	5010	1.69	1.49	1.92	2.32	1.9	2.82	-	-	-
				6975	1.88	1.5	2.35	2.6	1.86	3.64	-	-	-
Nakamura et al. (31)	2014	Japan	RCS	621	2.29	1.03	5.1	3.97	1.9	8.28	2.86	1.62	5.04
Packer et al. (32)	2021	USA	RCT	3636	1.71	1.22	2.41	1.58	1.11	2.68	1.91	1.27	2.86
Pang et al. (33)	2019	USA	PCS	499	-	-	-	-	-	-	-	-	-
Pokharel et al. (34)	2017	USA	PCS	448	2.29	1.49	3.52	-	-	-	-	-	-
Raymondi et al. (29)	2019	Spain	PCS	602	-	-	-	-	-	-	-	-	-
Roos et al. (35)	2022	Sweden	PCS	12869	1.78	1.5	2.11	1.75	1.35	2.26	-	-	-
Roset et al. (36)	2020	Spain	PCS	3190	2.67	1.7	4.19	-	-	-	-	-	-
Ruperti et al. (37)	2024	Switzerland	RCT	196	1.07	1.003	1.15	-	-	-	-	-	-
Suzuki et al. (39)	2019	Japan	PCS	155	-	-	-	1.02	1.01	1.03	-	-	-
Tentzeris et al. (40)	2014	Austria	PCS	188	1.81	1.07	3.05	4.54	1.92	10.73	3	1.71	5.24
Vigen et al. (6)	2024	USA	PCS	2971	1.17	1.06	1.29	1.07	0.86	1.26	-	-	-
Wijk et al. (38)	2015	Switzerland	RCT	622	2.53	1.76	3.64	-	-	-	-	-	-
Zhang et al. (41)	2023	China	PCS	1137	2.47	1.49	4.08	2.94	1.61	5.38	-	-	-

**Table 2:** The number of patients included for each outcome

**Total N**	**All-Cause Mortality**				**CV-Mortality**				**CV-Hospitalization**			
	**Low hs-cTnT**		**High hs-cTnT**		**Low hs-cTnT**		**High hs-cTnT**		**Low hs-cTnT**		**High hs-cTnT**	
	*event*	*N*	*event*	*N*	*event*	*N*	*event*	*N*	*event*	*N*	*event*	*N*
114,939	810	10861	2694	10427	229	10423	1482	11513	1501	6201	3433	10308
	7.4%		25.8%		2.2%		12.8%		24.2%		33.3%	

hs-cTnT: high sensitivity troponin T; CV: Cardiovascular

**Fig. 2:**
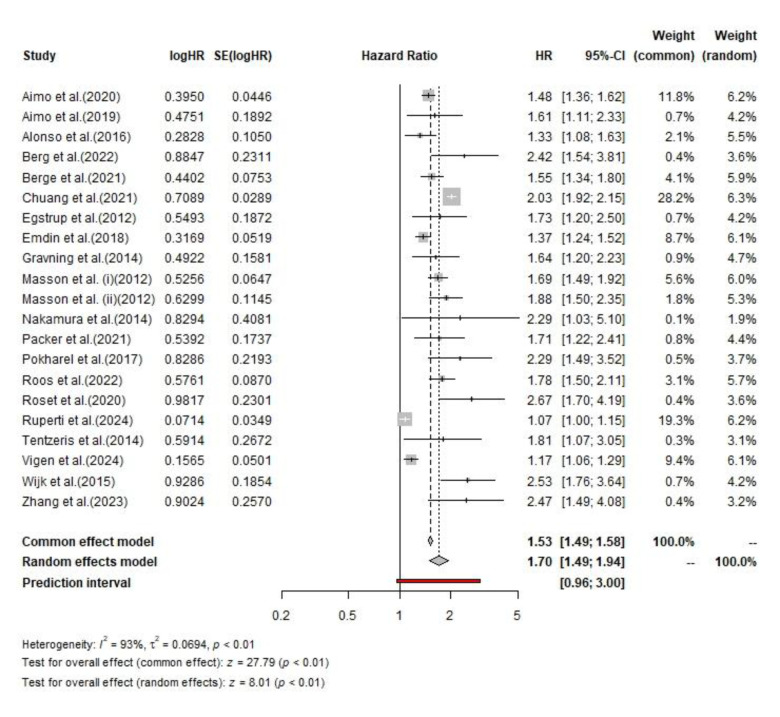
The pooled HR of all-cause mortality among CHF patients.

**Fig. 3:**
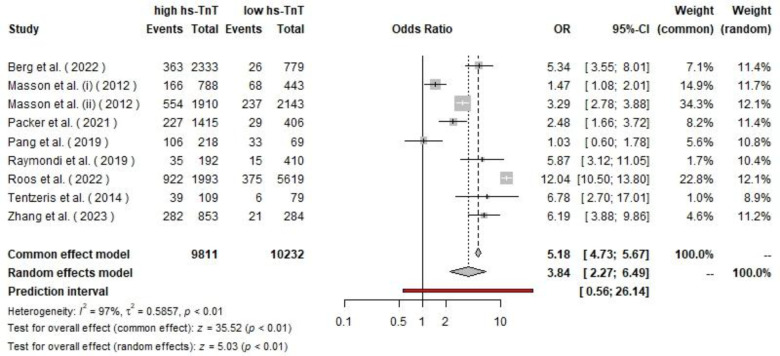
The pooled ORs of all-cause mortality among CHF patients

**Fig 4:**
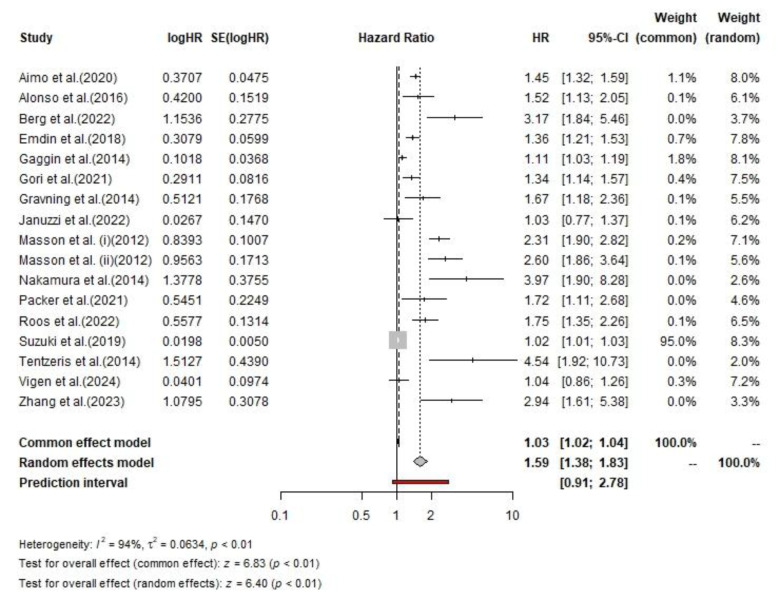
The pooled HRs of cardiovascular mortality among CHF patients

**Fig 5:**
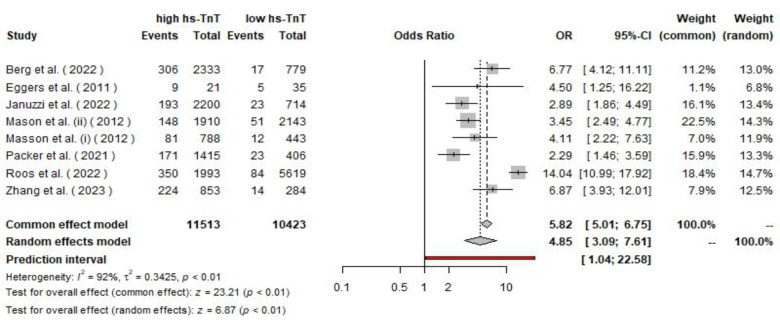
The pooled ORs of cardiovascular mortality among CHF patients

**Fig. 6:**
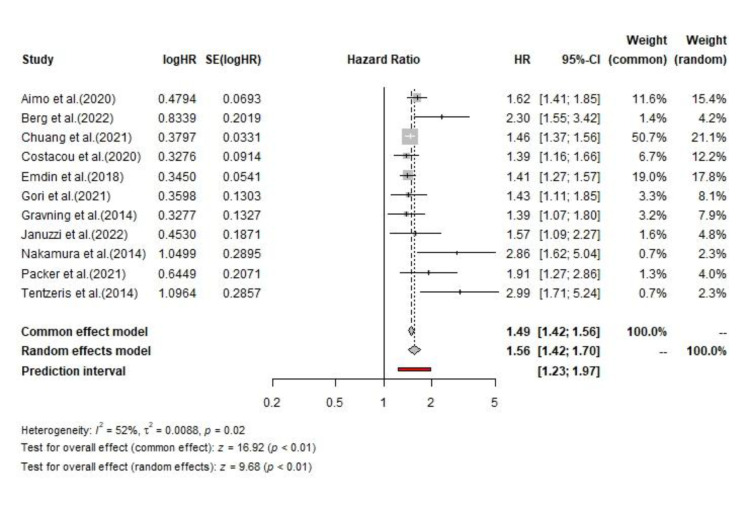
The pooled HRs of cardiovascular hospitalization among CHF patients

**Fig. 7:**
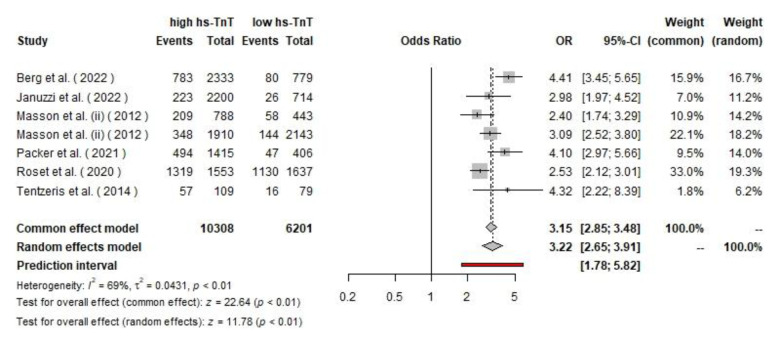
The pooled ORs of cardiovascular hospitalization among CHF patients

**Fig 8:**
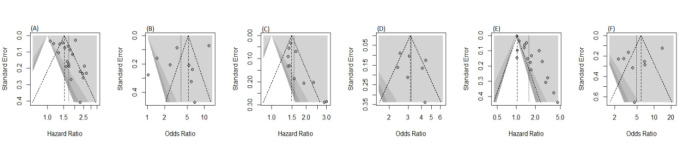
The funnel plots regarding publication bias assessment

(A) all-cause mortality HR; (B) all-cause mortality OR; (C) cardiovascular hospitalization HR; (D) cardiovascular hospitalization HR; (E) cardiovascular mortality HR; (F) cardiovascular mortality OR

## Discussion

This systematic review and meta-analysis aimed to evaluate the association between high-sensitivity troponin T (hs-cTnT) levels and clinical outcomes, including all-cause mortality, cardiovascular mortality, and cardiovascular hospitalization in patients with congestive heart failure (CHF). The findings demonstrated that elevated hs-cTnT levels were strongly associated with increased risks of adverse outcomes. Specifically, the pooled hazard ratios were 1.70 (95% CI, 1.49–1.94) for all-cause mortality, 1.59 (95% CI, 1.38–1.83) for cardiovascular mortality, and 1.56 (95% CI, 1.42–1.70) for cardiovascular hospitalization. Similarly, the pooled odds ratios were 6.19 (95% CI, 3.88–9.86), 6.87 (95% CI, 3.93–12.01), and 4.32 (95% CI, 2.22–8.39) for these respective outcomes. These results highlight the significant prognostic value of hs-cTnT in predicting adverse events in CHF patients, underscoring its potential utility in risk stratification and clinical decision-making.

Troponin T (TnT) is an essential regulatory protein found in cardiac muscle cells, playing a key role in myocardial contraction. Its measurement, particularly using high-sensitivity assays, has become integral to the diagnosis and management of cardiovascular diseases. Elevated levels of hs-cTnT reflect myocardial injury and correlate with the extent of cardiac damage. In the context of acute myocardial infarction (MI), hs-cTnT is a highly sensitive and specific marker that rises shortly after myocardial injury and remains elevated for several days, allowing for timely diagnosis and estimation of infarct size (6, 9).

Beyond acute MI, hs-cTnT also serves as a valuable prognostic marker in chronic cardiovascular conditions such as heart failure. This applies to both heart failure with reduced ejection fraction (HFrEF) and preserved ejection fraction (HFpEF) (37, 42). Even in the absence of acute ischemic events, elevated hs-cTnT levels offer insights into disease severity and progression, enabling clinicians to identify high-risk patients who may benefit from more aggressive therapeutic interventions. In clinical practice, hs-cTnT has shown utility in risk stratification, aiding in the prediction of outcomes such as cardiovascular death and hospital readmissions (43, 44).

The usefulness of hs-cTnT extends beyond heart failure and MI. Elevated hs-cTnT levels are frequently observed in conditions such as atrial fibrillation, pulmonary embolism, and chronic hypertension, indicating myocardial strain or subclinical injury (45–47). In pulmonary embolism, for instance, hs-cTnT reflects right ventricular strain, while in chronic hypertension, persistent elevation may indicate left ventricular hypertrophy or chronic myocardial stress, even in asymptomatic individuals. In such cases, hs-cTnT serves not only as a diagnostic biomarker but also as an indicator of long-term prognosis, including risks of heart failure progression and cardiovascular death (14, 28, 48).

The prognostic significance of hs-cTnT in heart failure is supported by accumulating evidence. This meta-analysis, which included over 21,000 patients, confirms the association between elevated hs-cTnT levels and adverse outcomes, such as increased all-cause mortality, cardiovascular mortality, and hospitalization. These findings are consistent with several other large-scale studies (45, 49–51). For example, in patients with nonischemic HFrEF, those with hs-cTnT levels above 21.5 ng/L experienced a 50% mortality rate over 30 months, compared with 14% among those with lower levels. Another study involving more than 200,000 individuals found that each one standard deviation increase in baseline hs-cTnT was associated with a 23% higher risk of all-cause mortality. Similarly, in acute heart failure, hs-cTnT levels above 35 ng/L were associated with nearly a threefold increase in the hazard of 30-day all-cause mortality. These results further validate hs-cTnT as a reliable prognostic marker in diverse HF populations.

In addition, the applicability of hs-cTnT extends to patients with comorbidities. In a study involving patients with both diabetes and heart failure, elevated hs-cTnT levels were significantly associated with major adverse cardiovascular events. This finding reinforces the broader clinical utility of hs-cTnT beyond traditional heart failure cohorts and supports its integration into routine evaluation, especially in patients with overlapping chronic conditions (45, 49–51).

Despite its value, a key challenge lies in the variability of cut-off values used to define elevated hs-cTnT levels across studies. This inconsistency complicates inter-study comparisons and may limit the clinical application of unified thresholds. Factors contributing to this variability include differences in patient populations, assay methodologies, and clinical settings (52–54). In most cases, cut-off values are used to dichotomize troponin levels into elevated versus normal, with higher levels indicating greater cardiovascular risk. However, determining a universally applicable threshold for predicting specific outcomes—such as death, hospitalization, or disease progression—remains complex. For instance, a 2023 study on nonischemic HF patients identified 21.5 ng/L as a critical cut-off linked to 50% mortality over 30 months, whereas in acute care settings, cut-offs such as 35 ng/L are used to indicate immediate risk (55–58).

Assay methodology is another significant contributor to this variability. The type and sensitivity of the assay used can affect both the absolute hs-cTnT values and the interpretation of results. In clinical practice, balancing sensitivity and specificity is essential. Lower cut-off values may improve sensitivity, capturing a broader range of at-risk patients but increasing false positives, while higher cut-offs improve specificity but may miss patients with subclinical injury. Therefore, optimal cut-off values must be context-dependent, reflecting the clinical scenario, population characteristics, and the intended use—whether diagnostic or prognostic (18, 35, 46).

Several limitations of this meta-analysis must be acknowledged. First, the substantial heterogeneity across included studies (with I² values ranging from 52% to 97%) reflects variations in study populations, designs, and clinical practices, potentially limiting the generalizability of the findings. Second, there is a possibility of publication bias, as studies reporting significant associations between hs-cTnT and outcomes are more likely to be published and included. This may lead to an overestimation of the true effect size. Finally, the lack of standardization in hs-cTnT assay techniques and cut-off definitions across studies poses a significant limitation, making it difficult to establish universally applicable clinical thresholds. Future research should focus on standardizing assay protocols and stratifying analyses based on patient subgroups, such as those with differing ejection fractions, comorbidities, or disease severity, to refine the prognostic utility of hs-cTnT in heart failure.

## Conclusion

This systematic review and meta-analysis demonstrates that elevated high-sensitivity cardiac troponin T (hs-cTnT) levels are strongly associated with adverse clinical outcomes in patients with heart failure (HF), including increased all-cause mortality, cardiovascular mortality, and cardiovascular hospitalizations. These findings support the role of hs-cTnT as a valuable prognostic biomarker, reinforcing its utility in risk stratification and early identification of high-risk patients who may benefit from intensified therapeutic strategies. The consistency of these associations across diverse HF populations underscores the broad clinical applicability of hs-cTnT in optimizing patient management.

Nevertheless, the substantial heterogeneity among the included studies, variability in assay methodologies, and potential biases necessitate cautious interpretation of the results. Future research should focus on standardizing hs-cTnT measurement protocols, investigating the influence of patient characteristics and comorbidities, and assessing its predictive value in specific HF subgroups to enhance its clinical relevance. Incorporating hs-cTnT testing into routine clinical practice has the potential to improve prognostic accuracy, inform therapeutic decision-making, and ultimately contribute to better outcomes in patients with heart failure. 
